# Production, Absorption, and Blood Flow Dynamics of Short-Chain Fatty Acids Produced by Fermentation in Piglet Hindgut during the Suckling–Weaning Period

**DOI:** 10.3390/nu10091220

**Published:** 2018-09-03

**Authors:** Masako Nakatani, Ryo Inoue, Shozo Tomonaga, Kikuto Fukuta, Takamitsu Tsukahara

**Affiliations:** 1 Department of Agricultural and Life Sciences, Kyoto Prefectural University, Hangi-cho, Shimogamo, Sakyo-ku, Kyoto 606-8522, Japan; ag8teff@gmail.com (M.N.); r-inoue@kpu.ac.jp (R.I.); 2 Laboratory of Nutritional Science for Animals, Division of Applied Biosciences, Graduate School of Agriculture, Kyoto University, Kitashirakawa Oiwake-cho, Sakyo-ku, Kyoto 606-8502, Japan; shozo@kais.kyoto-u.ac.jp; 3 Technical Center, Toyohashi Feed Mills, Kawada, Shinshiro, Aichi 441-1346, Japan; k-hukuta@toyohashi-shiryo.co.jp; 4 Kyoto Institute of Nutrition & Pathology, Furuikedani, Tachikawa, Ujitawara-cho, Kyoto 610-0231, Japan

**Keywords:** short-chain fatty acid, hindgut fermentation, absorption from lumen, suckling, weaning

## Abstract

Luminal short-chain fatty acids (SCFA) are rapidly absorbed from the intestine and subsequently utilized by the host as substrate for metabolic energy production. In pigs, the energy contribution of SCFA is thought to be 30–76%. However, since absorption and blood flow dynamics of SCFA in pigs, particularly during the suckling–weaning period, remain unclear, we aimed to elucidate these phenomena. Thirty-two piglets were used in the present work. Cecal vein blood and digesta, and portal and abdominal vein blood were sampled from suckling (7-, 14-, 21- and 28-day-old) and weaned (weaning at 21 and 28 days of age) piglets. Four piglets from each group were euthanized. SCFA concentrations in blood samples were analyzed by a highly sensitive gas chromatography-mass spectrometry technique. Age at weaning tended to affect SCFA absorption. For example, acetate and propionate concentrations in the cecal vein tended to be higher in piglets weaned at day 21 than at day 28. SCFA concentrations in the abdominal vein tended to differ from those in other veins. Mucosal gene expression analysis suggested that monocarboxylate transporter 1 and occludin were associated in absorption of SCFA from the lumen into the blood of piglets.

## 1. Introduction

Short-chain fatty acids (SCFA), particularly acetate, propionate and *n*-butyrate, are major end-products of gut microbiota [1]. SCFA production depends not only on substrates flowing into the large intestine [1], but also on the organ size and the population and composition of luminal bacteria [2]. Therefore, the large intestine is the major production site of SCFA in hindgut fermenters such as humans and pigs [2]. It is well established that luminal SCFA are rapidly absorbed from the intestine, and subsequently utilized by the host as substrate for metabolic energy production [3]. The energy contribution of SCFA to the basal metabolic rate is thought to be 30–76% [3] and 10% [4] in pigs and humans, respectively. Furthermore, SCFA are useful to the host not only for maintenance of the gut morphology and function [5,6] but also for reduction of appetite and diet-induced obesity [7,8].

During suckling, newborn mammals are fed only maternal milk. In humans [9] and pigs [10], maternal milk contains large amounts of disaccharides and oligosaccharides and thus, hindgut fermentation starts merely a few days after delivery [11]. When hindgut fermentation starts, the concentrations of SCFA in infant feces are approximately 60 mmol/kg [11]. Weaning, which occurs for all newborn mammals, can be a very stressful event. For example, it has been observed that the intestinal structure and function of piglets are drastically affected by weaning [12,13]. Moreover, during weaning, the transition from maternal milk to solid food affects not only the structure and function of the intestine but also the components of the luminal environment such as the intestinal microbiota and its metabolites. In pigs, it has been reported that weaning affects the luminal SCFA concentrations and composition in the large intestine [14,15]. Although the absorption process and blood flow dynamics of SCFA in weaning piglets remain unclear, most SCFA (95%) produced by the luminal microbiota is thought to be quickly absorbed from the mucosa, while only 5% is excreted in the feces [16]. van Beers-Schreurs et al. [17] compared the concentrations of SCFA in the portal and peripheral blood with those in the large intestinal digesta in weaning piglets. They reported that while giving a solid diet during weaning increased the SCFA concentrations in portal blood, the concentrations of SCFA remained unchanged in the intestinal lumen [17]. These data suggest that the concentrations of luminal SCFA do not always reflect the concentrations of absorbed SCFA.

Age at weaning age is one of the important factors for a good post-weaning development of pigs. In our previous study, early weaning at 14 days of age caused functional and morphologic ateliosis in piglets [12,13]. It is worth noting that marked maturation of the mucosal structure and function took place in 14- and 21-day-old suckling piglets [12]. Therefore, it is likely that age at weaning may also affect the ability of the intestine to absorb SCFA.

The aim of the present study was to evaluate the production and influx of SCFA in the cecum of pigs from suckling to weaning using a highly sensitive gas chromatography-mass spectrometry (GC-MS) technique [18]. Gene expression of SCFA transporters and molecules related to the tight junction were also assessed to elucidate the mechanism of SCFA absorption from the lumen into blood of piglets.

## 2. Materials and Methods

### 2.1. Animals

The 32 piglets used in the present study are shown in Table 1. The crossbred (Landrace × Large white × Duroc) piglets used in the present experiment were the same as those described in a previous study [13], except for piglets weaned at 14 days of age, which were omitted in the present work because we demonstrated that weaning at such age is not commercially practical for the pig industry [12,13]. All piglets were raised at the Toyohashi Feed Mills Technical Center (Shinshiro, Aichi, Japan). Suckling piglets were fed only maternal milk and weaning piglets were given a typical commercial weaning diet (JustOne Sprout; Toyohashi Feed Mills, Aichi, Japan). The nutrient composition of the diet was as follows (g/kg): crude protein, 214; crude fat, 75; crude fiber, 3; and crude ash, 60. All diets and water were given ad libitum. The animals were handled in accordance with the guidelines for animal studies of the Experimental Animal Committee of Kyoto Prefectural University (approval number KPU240410).

### 2.2. Dissection and Sampling

Ad libitum feeding was maintained until just before the dissection in all piglets. At the dissection, pigs were intraperitoneally anesthetized with sodium pentobarbital (Somnopentyl; Kyoritsu, Tokyo, Japan). All dissections started at 11:00 a.m. and collection of blood samples from all location of a piglet were finished within 5 min after confirmation of deep anesthesia. Briefly, the abdominal wall of pigs was incised along midline, blood was quickly collected from the cecal, portal, and abdominal veins, and the animals were euthanized by exsanguination. Afterward, the entire intestine was removed, the large intestine separated, and the cecal digesta collected. The cecum was washed several times with sterilized saline, and its middle section of mucosa soaked in RNA-later^®^ solution (Sigma, Tokyo, Japan), and stored first at 4 °C for 24 h, then at −80 °C until use. Blood samples were centrifuged at 1750× *g* for 10 min at 4 °C and serum was collected. Serum and digesta samples were stored at −80 °C until use.

### 2.3. Short Chain Fatty Acid Analysis by Ion-Exclusion High-Perfornance Liquid Chromatography

The concentrations of SCFA in the cecal digesta were measured by ion-exclusion high-performance liquid chromatography (HPLC) as previously described [18].

### 2.4. High-Sensitivity Detection of Short Chain Fatty Acid by Gas Chromatography-Mass Spectrometry

SCFA in serum samples were analyzed by GC-MS using a high-sensitivity detection method as previously described [18].

### 2.5. Gene Expression Analyses Using Real-Time Polymerase Chain Reaction

Total RNA extraction from cecal mucosa was conducted as described elsewhere [19]. cDNA synthesis and real-time polymerase chain reaction (PCR) were conducted as previously described by Inoue et al. [20]. Gene expressions of monocarboxylate transporter 1 (MCT1), sodium monocarboxylate transporter 1 (SMCT1), and occludin were evaluated by ∆∆*Ct* methods with reference to *glyceraldehyde 3-phosphate dehydrogenase* (*gapdh*) as internal control [21]. The primers and Taqman probes used in the present study are listed in Table 2.

### 2.6. Statistical Analyses

Depending on the results of the Bartlett test, either a complete randomized design, one-way analysis of variance (ANOVA) or the Kruskal–Wallis test was used to analyze the differences in each variable between S7, S14, S21, S28, W21p7, W21p14, W28p7, and W28p14. Tukey–Kramer post hoc (parametric or non-parametric) methods were used for multiple comparisons as needed. Correlation coefficient and its probability between parameters were analyzed by the Pearson’s correlation coefficient test. Differences between means were considered significant at *p* < 0.05. Values are given as the means ± standard errors. All data were analyzed using STATCEL4 (OMS, Saitama, Japan), an add-in package for Excel^®^ (Microsoft Corp., Redmond, WA, USA).

## 3. Results

### 3.1. Concentrations of Short-Chain Fatty Acids in the Cecal Digesta

**Figure 1:**
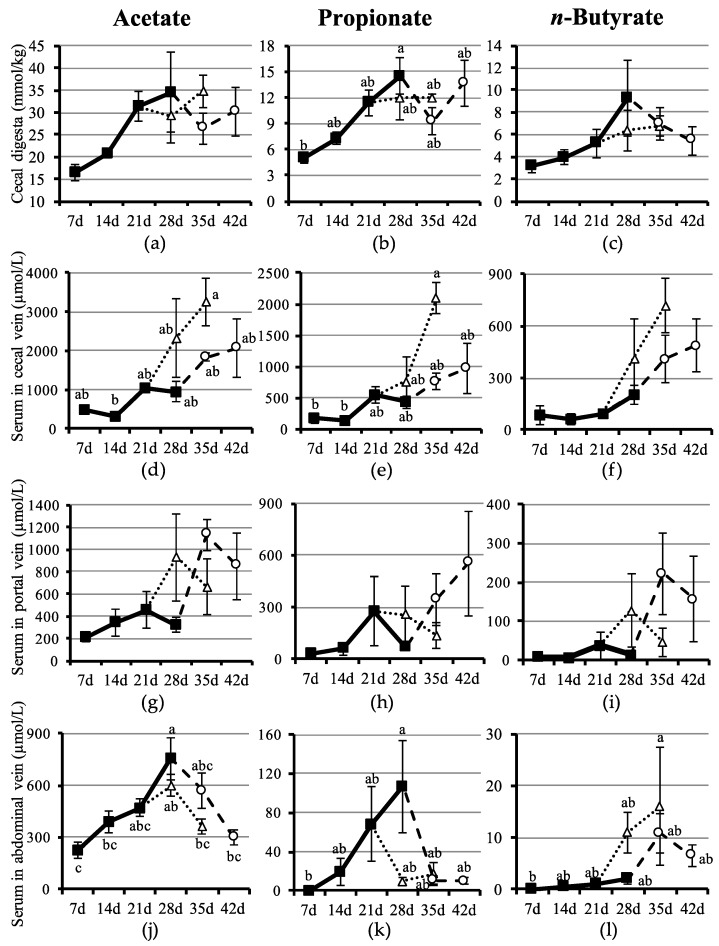
Short-chain fatty acid concentration in the cecal digesta, cecal vein, portal vein and abdominal vein of the piglets. (**a**) Concentration of acetate in the cecal digesta. (**b**) Concentration of propionate in the cecal digesta. (**c**) Concentration of *n*-butyrate in the cecal digesta. (**d**) Concentration of acetate in serum in the cecal vein. (**e**) Concentration of propionate in serum in the cecal vein. (**f**) Concentration of *n*-butyrate in serum in the cecal vein. (**g**) Concentration of acetate in serum in the portal vein. (**h**) Concentration of propionate in serum in the portal vein. (**i**) Concentration of *n*-butyrate in serum in the portal vein. (**j**) Concentration of acetate in serum in the abdominal vein. (**k**) Concentration of propionate in serum in the abdominal vein. (**l**) Concentration of *n*-butyrate in serum in the abdominal vein. Symbology: closed squares, suckling piglets; open circles, piglets weaned at 28 days of age; open triangles, piglets weaned at 21 days of age. Error bars represent the standard errors. Symbols with different letters (a, b and c) indicate significant differences at *p* < 0.05.

Acetate, propionate and *n*-butyrate concentrations were found to increase in the cecal digesta of piglets (Figure 1a–c), from day 7 to day 28 after birth, but only the concentration of propionate in S28 piglets was found to be significantly higher than that in S7 piglets (Figure 1b).

Age at weaning tended to affect SCFA concentrations in the cecal digesta. For example, although the concentrations of acetate and propionate were unaffected after weaning at day 21, they tended to be affected after weaning at day 28. The concentrations of acetate and propionate detected at weaning at day 28 temporarily decreased within the next seven days (W28p7), before recovering at day 14 post-weaning (W28p14). Nonetheless, these changes in concentrations were not significantly different.

### 3.2. Concentrations of Short-Chain Fatty Acids in the Cecal Vein

In serum in the cecal vein, SCFA concentrations did not change from day 7 to day 28 after birth, although weaning tended to increase the concentrations of acetate, propionate, and *n*-butyrate (Figure 1d–f). Particularly, acetate and propionate concentrations in W21p14 piglets increased significantly than those in S14 piglets.

### 3.3. Concentrations of Short-Chain Fatty Acids in the Portal Vein

In serum in the portal vein, changes in SCFA concentration were not detected (Figure 1g,h,j), due to the wide range of individual values.

### 3.4. Concentrations of Short-Chain Fatty Acids in the Abdominal Vein

The concentrations of SCFA in serum in the abdominal vein tended to differ from those in other veins (Figure 1j–l). For example, the concentrations of acetate and propionate gradually increased from day 7 onward, being those detected at day 28 significantly higher than those at day 7. It is worth noting that the concentration of *n*-butyrate was barely detected from day 7 to day 28 after birth (Figure 1l).

Age at weaning also tended to affect SCFA concentrations in the abdominal vein after weaning. For example, after weaning at day 21, the concentration of acetate initially tended to increase (W21p7), before showing a tendency to decrease at day 14 post-weaning (W21p14). In contrast, after weaning at day 28 a decrease in the concentration of acetate was detected, which by day 14 post-weaning (W28p14) became significant, when compared with S28 piglets. In addition, the concentration of propionate tended to decrease in piglets weaned at days 21 and 28. Regarding the concentration of *n*-butyrate, a non-significant increase was detected in all weaned piglets, regardless of age at weaning.

### 3.5. Gene Expressions of SCFA Transporters and Occludin in the Cecal Mucosa

**Figure 2:**
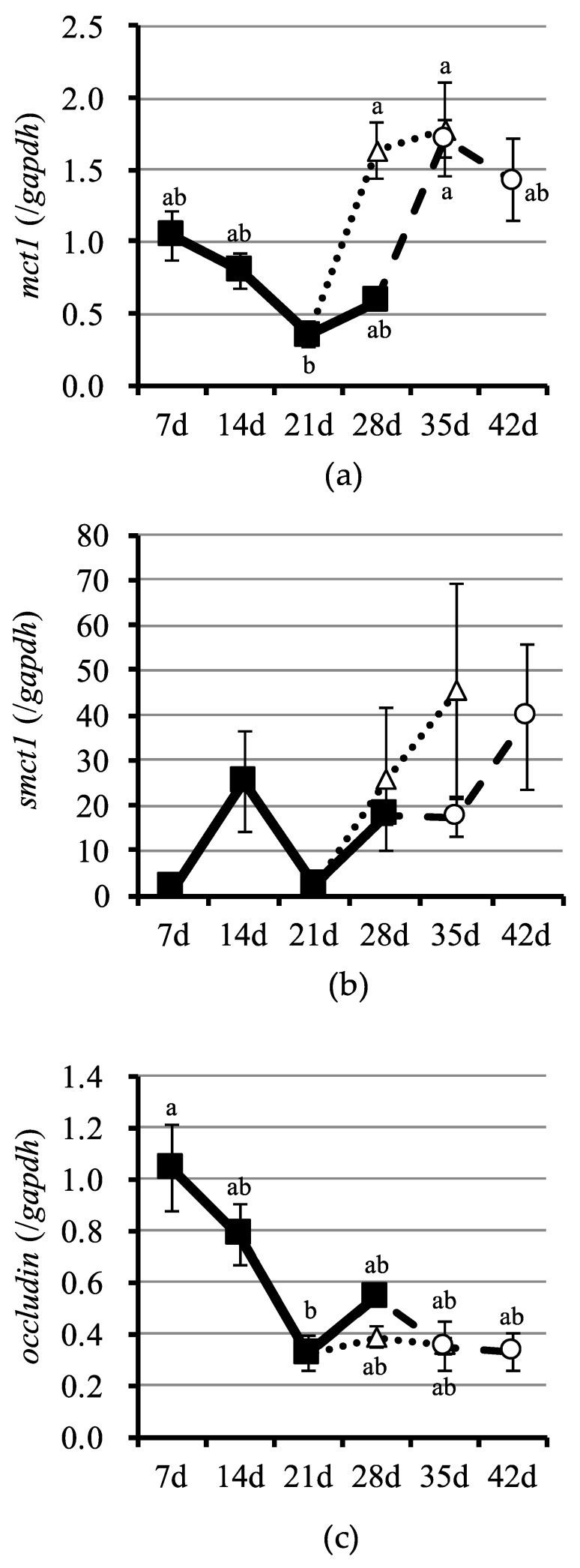
Relative gene expression of MCT1, SMCT1, and occludin in the cecal mucosa. (**a**) *Monocarboxylate transporter 1 (mct1)*. (**b**) *Sodium monocarboxylate transporter 1 (smct1)*. (**c**) *Occludin*. Symbology: closed squares, suckling piglets; open circles, piglets weaned at 28 days of age; open triangles, piglets weaned at 21 days of age. Error bars represent the standard errors. Symbols with different letters (a and b) indicate significant differences at *p* < 0.05.

Gene expression of SCFA transporters MCT1 and SMCT1, and occludin—a molecule related to the tight junction—is shown in Figure 2. While the gene expression of MCT1 decreased during suckling, it increased after weaning (Figure 2a). Indeed, there were significant differences between the gene expression of MCT1 observed at weaning days and after weaning (S21 vs. W21p7, W21p14, and W28p7). The highest expression of *smct1* was detected in S14 piglets (Figure 2b). In addition, *smct1* expression showed non-significant increases after weaning at days 21 and 28. Gene expression of occludin was the highest in S7 piglets, but it decreased over time (Figure 2c). While a significant difference was observed between S7 and S21 piglets, weaning did not affect the gene expression of occludin.

### 3.6. Correlation Analysis of Short-Chain Fatty Acids Concentration in the Cecal Vein

Correlations of the concentrations of SCFA in the cecal vein with those in other samples and gene expression associated with SCFA transporters and the tight junction are shown in Table 3.

While acetate and propionate concentrations in the cecal vein positively correlated with those in cecal digesta (*p* < 0.05), *n*-butyrate concentration in the cecal vein did not. Moreover, while the concentration of acetate in the cecal vein also positively correlated with that in the portal vein (*p* < 0.05), the concentrations of propionate and *n*-butyrate did not. Finally, the concentrations of SCFA in the cecal and the abdominal veins did not correlate. Interestingly, all SCFA concentrations in the cecal vein significantly correlated with the gene expression of MCT1 and occludin (*p* < 0.05). However, while the correlation with MCT1 gene expression was positive, the correlation with occludin gene expression was negative.

## 4. Discussion

SCFA are produced mainly in the hindgut of non-ruminant mammals [22]. In pigs, the cecum contains larger concentrations of SCFA in comparison with those found in the colon and rectum [6,22]. Therefore, in the present work cecum was chosen as the organ to evaluate SCFA production.

SCFA were readily detected in the cecal digesta of piglets, and their concentrations were detected to increase in suckling piglets from day 7 to day 28 after birth, but significant difference was not detected in acetate and *n*-butyrate (Figure 1a–c). Regarding the acetate, however, significant difference was observed between S14 and S21 when statistical analysis was performed on the values for only suckling piglets. Due to piglets ingested only maternal milk during suckling, the increase in SCFA concentrations in the cecal digesta may have been caused mainly by changes in milk composition and consequently in microbiota composition. For example, Noblet & Etienne [23] reported that the lactose concentration in milk gradually increases after parturition. Therefore, carbohydrates flowing into the cecum at higher concentrations are likely to further stimulate fermentation. The data from our previous study seem to validate this hypothesis because we showed that the gut microbiota of neonatal piglets undergoes changes even during suckling [24]. It has been well documented that weaning is one of the most stressful events for newborn mammals in general [25]. Indeed, the transition from liquid to solid food during weaning drastically affects the morphology and functions of the intestine [12,13], which in turn causes the gut microbiota to undergo profound changes [24]. In that context, in our study it was unexpected that SCFA concentrations in the cecal digesta did not change with weaning (Figure 1a–c). One possible explanation for the lack of change in SCFA concentration may be the size of cecum, which naturally increases after weaning [26]. The enlargement of cecum likely concealed an increase in the total amount of SCFA occurring in cecal digesta. Another fact that may help explain this unexpected outcome may be an increase in SCFA absorption after weaning, which is further discussed below.

SCFA found in blood serum in the cecal vein are those absorbed from the lumen following a partial utilization by the mucosal cells [27]. In the present work, absorption of SCFA was relatively low during suckling, but increased after weaning (Figure 1d–f). By contrast, although the concentrations of SCFA in the cecal vein did not change during suckling, they gradually increased in the cecal digesta during the same period (Figure 1a–c). These contrasting results seem to indicate that some sort of impaired absorption of SCFA took place in the lumen of suckling piglets. Nonetheless, it was detected that after weaning, SCFA concentrations in the cecal vein were high, which may indicate that absorption from the hindgut mucosa substantially increased in this period. When the correlation coefficient between the concentrations of SCFA in the cecal digesta and cecal vein were evaluated, it was found that acetate and propionate correlated positively, but *n*-butyrate did not (Table 3). These results seem to indicate that a considerable amount of *n*-butyrate was not absorbed into the cecal vein but remained within the cecal mucosa, perhaps utilized by the cells, especially the epithelial cells. The fact that *n*-butyrate is the major energy source of the epithelial cells in the large intestine [28] seems to validate our results. Interestingly, unlike in the suckling period, during the post-weaning period a higher concentration of *n*-butyrate was detected in the cecal vein (Figure 1f), which suggests that in weaned piglets, more *n*-butyrate flowed into blood and reached the liver through the portal vein (Figure 1i). In comparison, *n*-butyrate and other SCFA are transported through the portal vein to the liver of humans [29], implying that the dynamics of *n*-butyrate may be a similar feature in both humans and pigs.

When the host absorbs SCFA from the lumen, two routes have been observed: a ‘passive’ diffusion and an ‘active’ transport, but their contribution rate remains unclear [3], particularly during the suckling–weaning period. Passive diffusion, also known as gut permeability, permits the entry not only of SCFA but also of pathogenic microorganisms [30], which is generally problematic for the pig farming industry if it occurs after weaning [31]. The active transport of SCFA is a carrier-mediated transport dependent on metabolic energy, and it is believed that some transporters expressed by the epithelial cells are involved in the active transport of SCFA [32]. Indeed, HCO_3_/monocarboxylate exchange proteins, MCT and SMCT have been suggested to be facilitators of the influx of SCFA in the large intestine [16]. However, the HCO_3_/monocarboxylate exchange proteins in the large intestine are yet to be fully identified. For the present study, we selected two well-known transporters; MCT1 and SMCT1 as possible candidates involved in the active transport of SCFA in the epithelial cells of the intestine [16]. In the present work, although expression of *mct1* was induced by weaning (Figure 2a), that of *smct1* was not (Figure 2b). Moreover, *mct1* expression positively correlated with the concentrations of acetate, propionate and *n*-butyrate in the cecal vein (Table 3), suggesting that MCT1 plays an important role as active transport in suckling-weaned SCFA absorption. With regard to the passive diffusion of SCFA, weaning seems to increase gut permeability, because the expression of genes associated with the tight junction such as occludin, zonula occludens protein-1 and claudin-1 are downregulated by weaning [30,31]. Indeed, a downregulated expression of these genes induces an increased passive diffusion in a lactulose/mannitol tolerance test [30]. In the present study, while gene expression of occludin decreased during suckling, the expression of this gene was unaffected by weaning (Figure 2c). Furthermore, *occludin* expression negatively correlated with the concentrations of acetate, propionate and n-butyrate in the cecal vein (Table 3), suggesting that passive diffusion may also contribute to SCFA absorption in developing piglets.

Although no significance was found, the dynamics of SCFA in the portal vein bore a resemblance to those in the cecal vein (Figure 1g–i). Conversely, the dynamics of the concentrations of acetate and propionate in the abdominal vein were different from those in other veins (Figure 1j,k). Acetate and propionate concentrations increased in the abdominal vein during suckling, but sharply decreased after weaning (Figure 1j,k). After flowing into the liver, SCFA are readily metabolized by hepatocytes [33]. For example, propionate is almost all metabolized to glucogenic and lipogenetic substrates in the liver, hence it is hardly detected in the abdominal vein afterward [33]. In the present study, however, a relatively high amount of propionate was still observed in the abdominal vein of 28-day-old suckling piglets (Figure 1k). Two scenarios can be proposed to explain this apparent discrepancy: (1) under-development prevents the liver from completely metabolizing propionate during suckling; and (2) additional acetate and propionate may have directly flown in from the lymph fluid via the thoracic duct. To corroborate this possibility, we confirmed that the lymph fluid of suckling piglets contained higher concentrations of acetate (125 µmol/L) and propionate (2.0 µmol/L) than did that of weaned piglets (acetate: 83 µmol/L; propionate: undetected). Therefore, it can be cautiously asserted that the concentrations of acetate and propionate in the lymph fluid likely contributed to an increase in the concentrations of these SCFA in the abdominal vein during suckling.

## 5. Conclusions

In the present study, the dynamics of SCFA production and influx in piglets were evaluated from suckling to weaning. It was observed that while that the concentrations of SCFA in the cecal digesta changed from day 7 to day 28 after birth (suckling), those in the cecal vein did not. Our results suggested that some sort of impaired absorption of SCFA took place during suckling. Moreover, while the concentrations of SCFA in the cecal digesta were unaffected by weaning, those in the cecal vein substantially increased after weaning. Based on these results, it can be hypothesized that absorption from the hindgut mucosa likely begins after weaning. Gene expression analysis seemed to support this hypothesis, as it was observed in the cecal mucosa that while SCFA transporter *mct1* was upregulated, *occuludin*—a protein associated with the tight junction—was downregulated. It may be concluded that the present work demonstrated that age at weaning affected SCFA absorption, especially an earlier weaning at 21 days of age rather than a later weaning at 28 days of age. Nonetheless, because limited numbers of piglets were used in this study, further study is needed to determine our hypothesis regarding the effect of weaning.

## Figures and Tables

**Table 1:** Piglets used in the present study (*n* = 4) ^1^.

Age (Days)	Nutrition Period	Mean Body Weight (kg)	Age at Weaning (Days)	Identification Code
7	Suckling	3.7	–	S7
14	Suckling	5.3	–	S14
21	Suckling	7.0	–	S21
28	Suckling	10.4	–	S28
28	Weaned	9.0	21	W21p7
35	Weaned	13.1	21	W21p14
35	Weaned	12.4	28	W28p7
42	Weaned	15.9	28	W28p14

^1^ Piglets used were the same as those described in a previous study [13], except for piglets weaned at 14 days of age, which were excluded in the present work.

**Table 2:** Primers using this study.

Gene	Sequences (5′-3′)	Accession No.	Probe Number ^1^
Solute carrier family 16 member 1 (SLC16A1; MCT1)	F: tttgacactctaggcaatcagg R: gatgagagagaacagttatcggaag	NM_001128445	14
Solute carrier family 5 member 8 (SLC5A8; SMCT1)	F: tgtttgctttggggattttg R: caattccgacccacaaagaa	NM_001291414	20
Occludin	F: ggctaggggtctaaactgagc R: ctcagtgggttgaaggatctg	NM_001163647	5
Glyceraldehyde 3-phosphate dehydrogenase (GAPDH)	F: gtgacactcactcttctacctttga R: tgacaaagtggtcgttgagg	AF017079	45

^1^ Listed probe numbers indicate the product number of the Universal ProbeLibrary Set by Roche Applied Science (Mannheim, Germany).

**Table 3:** Correlation analysis between short-chain fatty acids (SCFA) concentrations in the cecal vein and other variables.

SCFA in the Cecal Vein	Other Variables	Correlation Coefficient	Probability ^1^
Acetate	Cecal digesta	0.56	<0.001
	Portal vein	0.46	0.002
	Abdominal vein	0.13	0.39
	*mct1*	0.44	0.003
	*smct1*	−0.02	0.88
	*occludin*	−0.43	0.003
Propionate	Cecal digesta	0.40	0.01
	Portal vein	0.15	0.31
	Abdominal vein	−0.01	0.96
	*mct1*	0.48	0.001
	*smct1*	0.26	0.08
	*occludin*	−0.40	0.01
*n*-Butyrate	Cecal digesta	0.20	0.19
	Portal vein	−0.02	0.88
	Abdominal vein	−0.01	0.96
	*mct1*	0.40	0.01
	*smct1*	0.16	0.28
	*occludin*	−0.38	0.01

^1^ When probability was less than 0.05, we considered significant correlation was observed between the parameters.
